# Dependence on Autophagy for Autoreactive Memory B Cells in the Development of Pristane-Induced Lupus

**DOI:** 10.3389/fimmu.2021.701066

**Published:** 2021-07-16

**Authors:** Albert Jang, Robert Sharp, Jeffrey M. Wang, Yin Feng, Jin Wang, Min Chen

**Affiliations:** ^1^ Department of Pathology and Immunology, Baylor College of Medicine, Houston, TX, United States; ^2^ Immunobiology and Transplant Science Center, Houston Methodist Research Institute, Houston, TX, United States; ^3^ Department of Surgery, Weill Cornell Medical College, Cornell University, New York, NY, United States

**Keywords:** autophagy, memory B cells, autoreactive memory B cells, lupus, pristane induced lupus, autoantibody, glomerulonephritis, systemic autoimmunity

## Abstract

The production of autoantibodies by autoreactive B cells plays a major role in the pathogenesis of lupus. Increases in memory B cells have been observed in human lupus patients and autoimmune *lpr* mice. Autophagy is required for the maintenance of memory B cells against viral infections; however, whether autophagy regulates the persistence of autoantigen-specific memory B cells and the development of lupus remains to be determined. Here we show that memory B cells specific for autoantigens can be detected in autoimmune *lpr* mice and a pristane-induced lupus mouse model. Interestingly, B cell-specific deletion of Atg7 led to significant loss of autoreactive memory B cells and reduced autoantibody production in pristane-treated mice. Autophagy deficiency also attenuated the development of autoimmune glomerulonephritis and pulmonary inflammation after pristane treatment. Adoptive transfer of wild type autoreactive memory B cells restored autoantibody production in Atg7-deficient recipients. These data suggest that autophagy is important for the persistence of autoreactive memory B cells in mediating autoantibody responses. Our results suggest that autophagy could be targeted to suppress autoreactive memory B cells and ameliorate humoral autoimmunity.

## Introduction

Systemic lupus erythematosus (SLE) is characterized by profound autoantibody production and tissue destruction (1–4). Autoreactive B cells are critical for the initiation and progression of lupus (2, 5–8). Persistent autoantibody production in SLE patients suggests the involvement of humoral memory against autoantigens in disease progression (9–11). Memory B cells are long-lived cells that have lower thresholds for activation and can be rapidly activated to differentiate into antibody secreting cells (12, 13). Cellular origins of the autoantibodies in lupus have been linked to memory B cells (9–11). Dysregulation of memory B cells has been identified in SLE patients and lupus-prone *lpr* mice (14–17). Memory B cells are also associated with disease relapses in SLE patients after B cell-directed therapies (15, 18–23). However, whether autoreactive memory B cells are critical for the pathogenesis of lupus remains to be determined.

Autophagy is a cellular digestion process during which double-membraned autophagosomes sequester cytoplasmic components, followed by fusion with lysosome and subsequent degradation of sequestered materials (24, 25). Autophagy-related genes, such as *ULK1, Atg7, Atg5/12* and *LC3*, are required for the formation of autophagosomes (26). Autophagy can help to generate energy and nutrients to protect cell viability caused by nutrient deprivation or lack of growth factors (24, 27, 28). Autophagy also contributes to quality control of cellular proteins and organelles to protect cell survival (29). We have found that autophagy is important for the long-term survival of memory B cells to maintain memory response to viral infections (30, 31). Autophagy has also been found to be critical for the protection of CD4^+^ and CD8^+^ memory T cells, as well as long-lived plasma cells (32–37). Using mice with Atg5 conditionally deleted in B cells, several groups have found that antibody responses are significantly reduced during T cell dependent or independent antigen immunization, parasitic infection, and mucosal inflammation (35, 36, 38). Loss of autophagy in B cells did not affect conventional B2 cell development (30, 36), class switch recombination or germinal center formation (30, 31), but impaired the maintenance of long-lived plasma cells in the bone marrow (35, 36, 38). Together these data suggest that autophagy is essential for the persistence of long-term immunological memory and antibody production. Using CD21-cre-Atg5^f/f^ mice crossed with autoimmune *lpr* mice, loss of autophagy has been shown to reduce bone marrow plasma cells and autoantibodies (38). Elevated autophagy is found to be increased in B cells from lupus patients and autoimmune NZB/NZW mice (39). Deletion of Atg5 in B cells attenuates the development of autoantibody production, lymphocyte infiltration and mortality in toll-like receptor 7-transgenic mice (40). Autophagy genes, such as *ATG5*, has been reported to be associated with an increased risk of developing lupus (4, 41–45). Moreover, anti-malaria drugs chloroquine and hydroxychloroquine that have long been used to treat lupus can also suppress autophagy (46–48). Dysregulation of autophagy has been detected in SLE patients and lupus-prone mice (39–41, 43, 44, 49–51). Therefore, autophagy may play a critical role for autoimmune responses in lupus. However, memory B cells specific for autoantigens have not been formally shown in previous studies. Moreover, an essential role for autophagy in the persistence of these autoantigen-specific memory B cells has not been established.

Pristane-induced lupus is a well-established murine model of systemic lupus erythematosus (52, 53). Susceptibility to pristane-induced lupus among non-autoimmune prone mice is widespread. It has been reported that C57BL/6 or BALB/c mice can develop anti-nuclear autoantibodies and immune complex-mediated glomerulonephritis, as well as other SLE-like symptoms following a single dose injection of pristane (52, 53). In this study, we show that autoreactive memory B cells can be induced in a pristane-induced lupus mouse model. Autophagy deficiency in B cells abrogates pristane-induced autoantibody production and glomerulonephritis with B cell-specific knockout of Atg7. Moreover, adoptive transfer of wild type memory B restored autoantibody production in Atg7-deficient recipient mice. Our study suggests that autophagy is important for the persistence of autoreactive memory B cells, maintenance of autoantibody production, and sustained glomerulonephritis in pristane-induced lupus.

## Material and Methods

### Mice

Atg7^flox^ mice were obtained from Dr. Masaaki Komatsu of Tokyo Metropolitan Institute of Medical Science (54) and crossed with CD19-cre knock-in mice (The Jackson Laboratory) to obtain B/Atg7^–/–^ mice. *MRL* and *MRL*-*lpr* mice were obtained from the Jackson Laboratory. Sex and age-matched wild type or B/Atg7^–/–^ mice in the C57BL/6 background at the age of 8-12 weeks were used at the start of all experiments except noted. For pristane injection, healthy sex and age-matched B/Atg7^–/–^ mice and wild type controls (8-12 weeks old) were randomly separated into groups for pristane or phosphate-buffered saline (PBS) injection (as controls). A single dose of pristane (0.5 ml i.p.) was injected. Sera were collected 6 months post-injection and levels of autoantibodies were measured by ELISA. Mice were sacrificed at 6 months after pristane injection. Spleens were harvested for Fluorescence-Activated Cell Sorting (FACS) and kidneys and lungs were collected for histology analysis. At least 5 mice per group were used for each experiment. At least 10 mice per group were used for pristane injection experiments. The experiments were performed according to federal and institutional guidelines and with the approval of Institutional Animal Care and Use Committees of Baylor College of Medicine and Houston Methodist Research Institute.

### Antibodies

The following antibodies from BD Biosciences were used for flow cytometry: biotinylated antibodies to CD4 (553728), CD8 (553029), IgM (553436), CD11b (553309), CD138 (553713) and GR-1 (553125); PE-conjugated antibodies to B220 (553090), CD5 (553023), CD11b (557397), IgD (558597), CD138 (553714); APC-conjugated antibodies to CD21 (558658), IgM (550676), IgD (560868), CD11b (553312), CD138 (558626) and GR-1 (553129); FITC-conjugated antibodies to GL7 (553666), IgD (553439); PE-Cy7 conjugated anti-CD11b (552820); Pacific Blue anti-CD3e (558214); PE-Cy5-anti-CD4 (553050) and APC-Cy7-anti-CD8a (557654). From Biolegend: APC-anti-mouse IgG1 (406610), PerCPCy5.5-anti-mouse IgG1(406612), Pacific Blue anti-CD38 (102720), FITC-anti-mouse IgG (405305), APC-anti-mouse IgG (405308), APC-Cy7-anti-mouse IgG (405316). From eBioscience: PerCP-Cy5.5-anti-B220 (45-0452-82), PE-anti-IgM (12-5890-83), PE-anti-CD23 (12-0232-82), Biotin-anti-mouse-IgD (13-5993-85), Streptavidin-PE (12-4317-87), PE-Cy7-streptavidin (25-4317-82). From the Jackson Immunoresearch Laboratories: normal rabbit IgG (015-000-002) or mouse IgG (011-000-002). From Abgent: anti-LC3 (AP1802a) for immunocytochemistry. From Invitrogen: Anti-CoxIV (459600) for immunocytochemistry. From Southern Biotechnology, HRP conjugated anti-mouse IgG or IgM.

### Flow Cytometry

Spleens were treated with 0.4 mg/ml liberase (Roche) at room temperature for 10 min to make single cell suspension of splenocytes, followed by lysis of red blood cells with ACK lysis buffer. After blocking with 1 μg/ml anti-CD16/CD32, 10 μg/ml rat IgG and 10 μg/ml hamster IgG, the cells were then incubated with various antibodies conjugated to FITC, PE, APC, PerCP-Cy5.5, Pacific Blue (BD Biosciences) or PE-conjugated anti-PDCA-1 (Miltenyi Biotec) and analyzed by flow cytometry. Double-stranded DNA (dsDNA) were conjugated to Alexa fluor 488 using ULYSIS Nucleic Acid Labeling Kit (U21650, Invitrogen) according to manufacturer’s instructions. Autoantigen RNP/Sm (The Binding Site) was labeled with Alexa Fluor 488 using the Microscale Protein Labeling Kit (A30006, Invitrogen). To detect dsDNA-specific memory B cells and germinal center B cells, total spleen cells were stained with PE-conjugated antibodies to CD11b, IgM, IgD, GR1 and CD138 (DUMP), APC-anti-mouse IgG, PerCP-Cy5.5-anti-CD19 or B220, Pacific Blue anti-CD38 and Alexa Fluor 488-dsDNA. To detect RNP-specific memory B cells and GC B cells, total spleen cells were stained with PE-conjugated antibodies to CD11b, IgM, IgD, GR1 and CD138 (DUMP), APC-anti-mouse IgG, PerCP-Cy5.5-anti-CD19 or B220, Pacific Blue anti-CD38 and Alexa Fluor 488-RNP/Sm. DUMP^-^B220^+^IgG^+^dsDNA^+^CD38^+^ and DUMP^-^B220^+^IgG^+^RNP/Sm^+^CD38^+^ memory B cells, DUMP^-^B220^+^IgG^+^dsDNA^+^CD38^–^ and DUMP^-^B220^+^IgG^+^RNP/Sm^+^CD38^–^ germinal center B cells were analyzed by flow cytometry.

### Quantitative Real Time RT-PCR (qRT-PCR)

CD19^+^IgM^low^IgD^+^CD23^+^IgG^-^ naïve mature B cells, CD19^+^DUMP^-^IgG^+^CD38^+^dsDNA^+^ and CD19^+^DUMP^-^IgG^+^CD38^+^RNP/Sm^+^ memory B cells were sorted from 3-4 months old *lpr* mice. RNA extracted from the cells was used to prepare cDNA with the High Capacity cDNA Reverse Transcription Kit (Life Technologies). Real-time PCR was performed using Taqman Universal PCR Master Mix with specific primers for autophagy genes or 18S rRNA from the TaqMan Gene Expression Assay Kit (AB Applied Biosystem) in the ABI PRISM 7000 Sequence Detection System. The assay IDs for the primers of the analyzed genes are: Mm00504340_m1 (Atg5), Mm00512209_m1 (Atg7), Mm00437238_m1 (ULK1), Mm00458725_g1 (MAP1LC3A), Mm00782868_sH (MAP1LC3B), and Mm00553733_m1 (Atg14). Relative gene expression was normalized to 18S rRNA.

### Enzyme-Linked Immunosorbent Assay (ELISA)

To detect autoantibodies against autoantigens, 96-well plates coated with Sm, or RNP (The Binding Site) were incubated with serially diluted sera at 37°C for 2h. The plates were then washed and incubated with HRP-conjugated goat anti-mouse IgG (Southern Biotechnology, Birmingham, AL) at 37°C for 1h, followed by development with TMB peroxidase EIA substrate (Bio-Rad, Hercules, CA). The reaction was stopped with 1N H_2_SO_4_ and the optical density at 450 nm was measured using an ELISA reader. A mixture of sera from MRL-*lpr* mice was used to establish standard curves in each plate and antibody levels were shown as relative titers.

### ELISPOT

MultiScreen 96-well Filtration plates (Millipore) were coated with 2 μg/ml RNP+Sm (The Binding Site). Sorted RNP/Sm+ or nonspecific memory B cells (100, 1000, 10000/well) were activated with LPS (10ug/ml) for 3 days, then added to the plates and incubated at 37 °C for 5 h. The cells were lysed with H_2_O and the wells were probed with HRP-conjugated goat anti-mouse IgG (Southern Biotechnology), followed by development with 3-amino-9-ethylcarbzole (Sigma).

### Adoptive Transfer of Autoreactive Memory B Cells

Wild type mice which were injected with pristane 6 months earlier were used as donors of wild-type memory B cells. Sex and age-matched 8-12 week-old B/*Atg7* ^–/–^ and wild type mice were used as recipients of the memory B cells. DUMP^-^B220^+^IgG^+^CD38^+^RNP/Sm^+^ memory B cells were sorted from pooled spleens of pristane injected donor mice and adoptively transferred into wild type and B/Atg7^-/-^ recipient mice retrooribitally (10,000 cells/mouse). Sorted naïve cells (2x10^5^/mouse) from untreated mice were co-injected as filler cells to minimize cell loss during injection. One day after transfer recipient mice were injected with a single dose of 0.5 ml pristane i.p. Sera were collected 2 months post-injection. For some experiments, recipient mice were immunized with 20 μg RNP/Sm precipitated with 100 μl Inject Alum (Thermo Scientific) intraperitoneally (20 μg/mice, i.p.) and sera were collected 3 days later. Levels of autoantibodies in the sera of recipients were measured by ELISA.

### Histochemistry and Immunocytochemistry Staining

To detect immune complex deposits in the kidney, frozen sections of the kidneys were stained with FITC-conjugated goat anti-mouse IgG (Sigma) or FITC-conjugated goat anti-mouse complement C3 (MP Biochemical, 55500) and analyzed under a fluorescent microscope. Scores of glomerular IgG and complement C3 deposition were assigned based on the intensity of IgG/C3 deposition (range 0-3) with 0 represents no deposition and 3 representing intense depositions. Sections of kidney and lung were also stained with hematoxylin and eosin (H&E). The degree of glomerulonephritis was graded using a glomerulonephritis activity score (range 0–24) developed for the assessment of lupus nephritis in humans (55). Glomerular cells in 10 glomeruli per section were counted. We also measured anti-nuclear antibodies (ANAs) in pristane-treated mice by staining of Hep2 cells according to our described protocol (56). Hep-2 cells on slides (Medical and Biological Laboratories) were incubated with serially diluted sera followed by staining with FITC-conjugated anti-mouse IgG (Sigma). The staining was visualized under a BX-51 fluorescence microscope (Olympus). LC3 staining were performed according to our previously described protocol (30, 31). Briefly, sorted RNP-specific memory B cells from MRL-*lpr* mice or pristane-treated wild type mice, and naïve B cells from untreated wild type mice were added to slides by cytospin. The cells were fixed, incubated with a rabbit antibody to processed LC3 (Abgent) and followed by staining with Alexa Fluor-conjugated secondary antibodies (Molecular Probes). The nucleus was counter-stained with DAPI. The cells were then analyzed using a SoftWorx Image deconvolution microscope (Applied Precision).

### Statistical Analyses

Data were presented as the mean ± SEM, and *P* values were determined by two-tailed Student’s *t*-test using GraphPad Prism software and are included in the figure legends. The comparison of survival curves between and WT and KO was performed by Log-rank (Mantel-Cox) test using Prism. Significant statistic differences (*P*<0.05 or *P*<0.01 or *P*<0.001 or *P*<0.0001) are indicated.

## Results

### Increased Memory B Cells Specific for Autoantigens in Autoimmune *lpr* Mice

**Figure 1:**
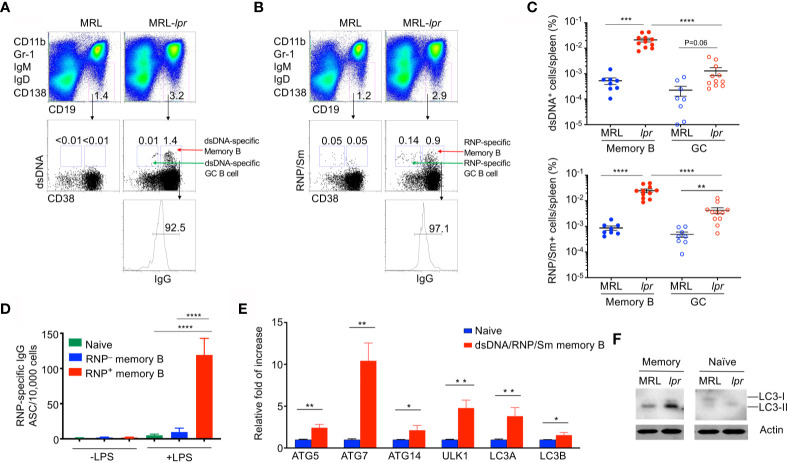
Elevated autophagy in autoantigen-specific memory B cells. Splenocytes from MRL or MRL-*lpr* mice (4-month-old) were stained with PE-conjugated antibodies to CD11b, Gr-1, IgM, IgD and CD138, PE-Cy7-anti-CD19, Pacific Blue anti-CD38, APC-anti-IgG, and Alexa Fluor 488-conjugated dsDNA or RNP. DUMP^–^ (IgM^–^IgD^–^CD11b^–^Gr-1^–^CD138^–^) CD19^+^ B cells were gated. **(A)** dsDNA-specific memory B cells (DUMP^–^CD19^+^CD38^+^dsDNA^+^IgG^+^) or **(B)** RNP-specific memory B cells (DUMP^–^CD19^+^CD38^+^RNP^+^IgG^+^) were analyzed by flow cytometry. **(C)** The frequency of dsDNA-specific memory B cells and RNP-specific memory B cells, as well as dsDNA-specific germinal center (GC) B cells (DUMP^–^CD19^+^CD38^–^dsDNA^+^IgG^+^) and RNP-specific GC B cells (DUMP^–^CD19^+^CD38^–^RNP^+^IgG^+^), in the spleen of each mouse was plotted. Data are presented as mean ± s.e.m. **P < 0.01, ***P < 0.001, ****P < 0.0001, (n=8 for MRL and 11 for MRL-*lpr*). **(D)** RNP-specific memory B cells (DUMP^–^CD19^+^CD38^+^RNP^+^IgG^+^) and RNP-negative memory B cells (DUMP^–^CD19^+^CD38^+^RNP^–^IgG^+^) were sorted from MRL-*lpr* mice and stimulated with LPS in *vitro* for 3 days, the number of ASCs producing RNP-specific antibodies were measured by ELISPOT. Data are presented as mean ± s.e.m. Experiments were performed twice in triplicates using cells from a pool of 3-4 mice. ****P < 0.0001, determined by two-tailed Student’s *t*-test. **(E)** Splenocytes from pooled MRL or MRL-*lpr* mice were stained as in **(A)** except that both Alexa Fluor 488-conjugated dsDNA and RNP were included for staining. Sorted dsDNA/RNP/Sm-specific memory B cells, and B220^+^IgM^low^IgD^+^CD23^+^IgG^−^ naïve B cells were used for real-time RT-PCR analysis of indicated autophagy-related genes. Data are presented as mean ± s.e.m. Experiments were performed three times using cells from a pool of 10-15 mice. *P < 0.05, **P < 0.01, determined by two-tailed Student’s *t*-test. **(F)** Western blot analysis of LC3 processing in Memory (DUMP^-^CD19^+^IgG^+^CD38^+^) and naïve B cells isolated from pooled MRL and MRL-*lpr* mice. Data are representative of two independent experiments.

Autoimmune Fas-deficient *lpr* mice develop significant lymphoproliferation and increased autoantibodies against DNA and nuclear antigens (7, 57–60). These mice also exhibit nearly 10-fold increase in the percentage of IgG1^+^ memory B cells (16, 17). We therefore determined whether memory B cells specific for autoantigens could be detected in *lpr* mice. Anti-dsDNA and anti-RNP/Sm autoantibodies are associated with lupus in both humans and mice (61, 62). We generated fluorochrome-conjugated dsDNA and RNP/Sm in order to stain B cells specific for these autoantigens. Interestingly, we detected dsDNA-specific CD19^+^IgG^+^IgD^–^IgM^–^CD38^+^ memory B cells in MRL-*lpr* mice (**Figure 1A**). In contrast, these dsDNA-specific autoantigen-specific memory B cells were absent in age-matched control MRL mice (**Figure 1A**). We also detected RNP/Sm-specific memory B cells in MRL-*lpr* mice but not in control MRL mice (**Figure 1A**). Moreover, these CD19^+^IgG^+^IgD^–^IgM^–^CD38^+^RNP/Sm^+^ memory B cells could be activated by LPS *in vitro* to differentiate into a larger number of anti-RNP/Sm antibody secreting cells (ASCs) than RNP/Sm-negative memory B cells could, suggesting the CD19^+^IgG^+^IgD^–^IgM^–^CD38^+^RNP/Sm^+^ memory B cells identified by flow cytometry are bona fide RNP/Sm-specific memory B cells (**Figure 1D**). We found that B cells specific for dsDNA and RNP/Sm are predominantly the CD19^+^IgG^+^IgD^–^IgM^–^CD38^+^ memory B cells, but not the CD19^+^IgG^+^IgD^–^IgM^–^CD38^–^ germinal center (GC) B cells (**Figures 1A–C**). This indicates that *lpr* mice contain abundant memory B cells but not germinal center B cells specific for autoantigens.

### Enhanced Autophagy in Memory B Cells From Lupus-Prone *lpr* Mice

We have previously observed increased autophagy in memory B cells (30). We therefore stained memory B cells with dsDNA and RNP/Sm and sorted these autoreactive memory B cells. We then examined whether these autoreactive memory B cells displayed increased autophagy gene expression. As shown by quantitative RT-PCR (qRT-PCR), autoreactive memory B cells also expressed increased levels of *Ulk1* (Atg1) and *Atg14* critical for autophagy initiation, as well as *Atg5*, *Atg7*, *Map1lc3a* and *Map1lc3b* that required for autophagosome maturation (63–65) (**Figure 1E**). These data suggest that autoreactive memory B cells from *lpr* mice display active autophagy.

The conversion from LC3-I to LC3-II isoforms is indicative of active autophagy (26, 66). We therefore performed Western blot analysis to detect LC3. In comparison with naïve B cells, memory B cells from wild type and *lpr* mice displayed increased levels of LC3-II (**Figure 1E**). Moreover, memory B cells from *lpr* mice displayed significantly increased LC3-II compared to wild type controls (**Figure 1F**). These data suggest that memory B cells from *lpr* mice display active autophagy.

### Autophagy Deficiency Inhibits Pristane-Induced Autoantibody Production

**Figure 2:**
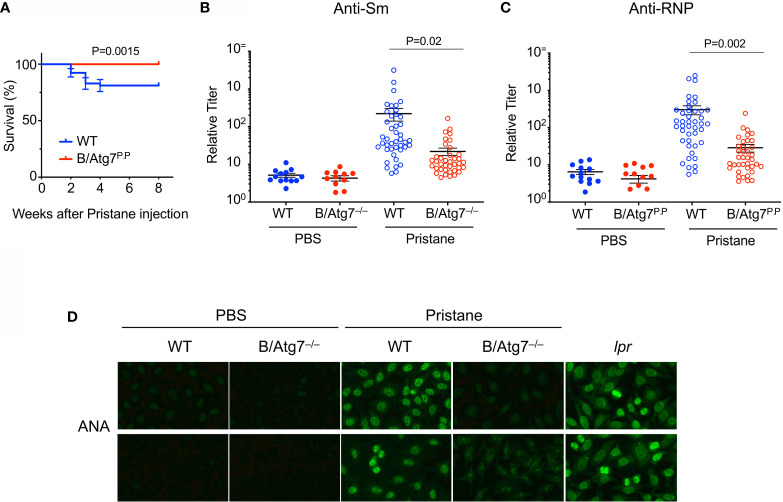
Decreased production of autoantibodies in B/Atg7^–/–^ mice injected with pristane. Sex and age-matched 8-12 weeks old B/Atg7^–/–^ and wild type mice were injected with a single dose of 0.5 ml pristane or PBS (i.p.). **(A)** Mouse survival after pristane injection was plotted. Data are combined results from three experiments. (n= 53 and 48 for WT and B/Atg7^–/–^ with pristane injection, respectively). The comparison of survival curves between and WT and KO was performed by Log-rank (Mantel-Cox) test using Prism. p = 0.0015. **(B, C)** Sera were collected 6 months later and levels of autoantibodies specific for Sm **(B)** or RNP **(C)** were measured by ELISA using plates coated with RNP or Sm (The Binding Site). Data are combined results from three experiments. (n=13 and 11 for WT and B/Atg7^–/–^ with PBS injection, respectively; n= 43 and 38 for WT and B/Atg7^–/–^ with pristane injection, respectively). **(D)** Anti-nuclear antibodies (ANAs) were detected by incubation of sera from WT (1:160 dilution) or B/Atg7^–/–^ mice (1:40 dilution) injected with pristane or PBS (1:40 dilution), B6-*lpr* (1:320 dilution) with Hep2 cell slides, followed by probing with FITC-conjugated anti-mouse IgG. Sera from 9/10 of WT and 1/10 of B/Atg7^–/–^ mice gave positive nuclear staining. Representative images from each group were shown (n=10 per group).

**Figure 3:**
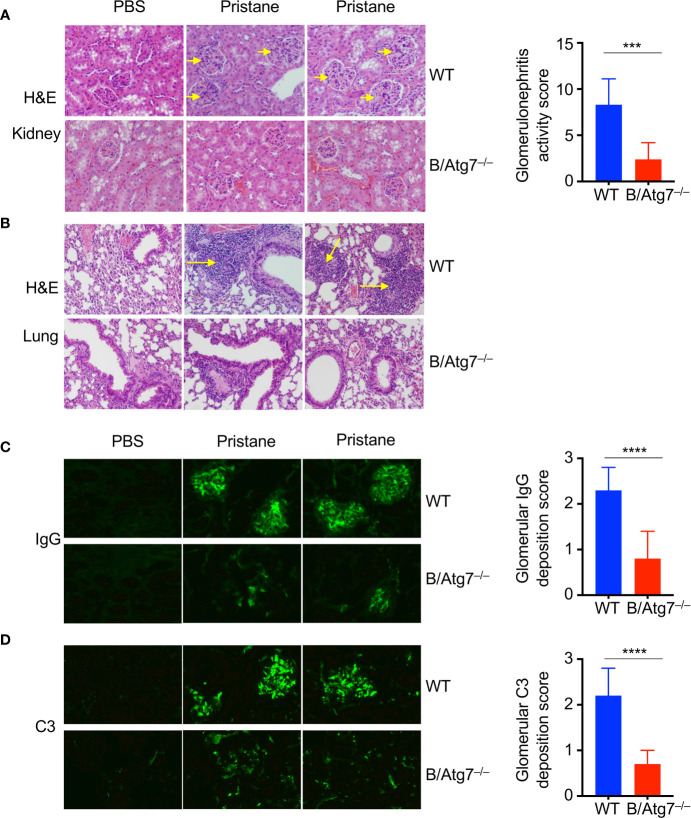
Induction of glomerulonephritis in pristane injected-wild type but not B/Atg7^–/–^ mice. H&E staining of kidney **(A)** and lung **(B)** sections of WT and B-Atg7**^–/–^** mice 6-month after injection with pristane or PBS. Enlarged glomeruli were marked by arrows in **(A)**. Lymphocyte infiltration was marked by arrows in **(B)**. Glomerulonephritis activity score was 8.3 ± 2.8 *versus* 2.4 ± 1.8 for wild type and B/Atg7^–/–^ mice, respectively. **(C)** Kidney sections were stained with FITC-anti-mouse IgG to reveal IgG deposit. **(D)** Kidney sections were stained with FITC anti mouse-complement C3. Glomerulonephritis deposition scores for IgG and C3 were plotted and presented as mean ± SD. ***P < 0.001, ****P < 0.0001, as determined by two-tailed Student’s *t*-test. Representative images from 2 mice each group were shown. (n=8 per group).

It has been shown that autophagy is elevated in SLE, and may be essential for humoral autoimmune manifestations (38, 39, 49). We therefore investigated whether autophagy might be important for the protection of autoreactive memory B cells and the production of autoantibodies using a pristane-induced lupus model. We crossed CD19-cre mice with Atg7^flox^ mice (54) to generate B cell-specific deletion of Atg7 (B/Atg7^–/–^) on the C57BL/6 background. B/Atg7^–/–^ and wild type mice were then administrated with pristane. Pulmonary hemorrhage resembling that seen in SLE has been reported to occur earlier in pristane-treated mice and causes mortality in a portion of mice on the C57BL/6 background (52, 53, 67). We observed that treatment with pristane caused 18% death in wild type mice within 4 weeks of pristane injection (**Figure 2A**). Interestingly, B/Atg7^–/–^ mice were resistant to pristane-induced death (**Figure 2A**). Presumably the mortality associated with pristane-induced pulmonary vasculitis (52, 53, 67) is prevented due to the loss of autophagy in B cells (also see **Figure 3B**).

It has been reported that pristane-induced lupus induces production of a different spectrum of SLE associated autoantibodies in C57/BL6 and BALB/c mice (52, 53). C57/BL6 mice could produce anti-RNP and anti-Sm (57, 62) but not anti-dsDNA autoantibodies after pristane treatment (52, 53). We next measured lupus-associated anti-dsDNA, anti-RNP and anti-Sm autoantibody levels by ELISA in sera from these mice six months after pristane administration. Consistent with previous reports, we did not detect significant induction of anti-dsDNA autoantibodies in wild type or B/Atg7^–/–^ mice on the C57/BL6 background (data not shown). We detected significant induction of anti-RNP and anti-Sm in wild type mice after pristane treatment (**Figures 2B, C**). However, the induction of anti-RNP and anti-Sm autoantibodies was dramatically reduced by 10-fold in B/Atg7^–/–^ mice (**Figures 2B, C**).

We also measured anti-nuclear antibodies (ANAs) in pristane-treated mice by staining of Hep2 cells. As a positive control, we detected distinct nuclear staining with sera from B6/*lpr* mice (**Figure 2D**). We detected nuclear staining of Hep2 cells with sera from 90% pristane-treated wild type mice (**Figure 2D**). Among them, 1/10 sera gave strong nuclear staining (1:640 dilution); 4/10 sera gave medium nuclear staining (1:160 dilution); and 4/10 sera gave low nuclear staining (1:40 or 1:80 dilution). Majority of the staining pattern is speckled (**Figure 2D**), which are typically produced by elevated level of anti-RNP/Sm autoantibodies. Sera from one mouse gave strong cytoplasmic staining (data not shown). In contrast, no significant staining was observed using sera from 8 of the 10 B/Atg7^–/–^ mice (**Figure 2D**). Only 1 of the 10 sera from B/Atg7^–/–^ mice generated detectable but much weaker speckled nuclear staining in Hep2 cells (1:40 dilution), whereas weak cytoplasmic staining was found in sera from 1 mouse. These results indicate that pristane induces the production of ANAs in wild type mice but loss of autophagy in B cells significantly reduced ANA production in B/Atg7^–/–^ mice.

### Autophagy Deficiency Inhibits Pristane-Induced Glomerulonephritis and Pulmonary Vasculitis

To determine whether deficiency in autophagy affects other manifestations of autoimmunity, we examined kidneys from mice treated with pristane. Pristane treatment has been shown to induce the development of glomerulonephritis in mice (52, 53, 68). Consistently, the sizes of glomeruli in the kidney sections from pristane-treated wild type were increased compared to untreated controls (**Figure 3A**). Moreover, increased number of intraglomerular mesangial cells were found in these wild type mice treated with pristane (**Figure 3A**). In contrast, pristane treatment did not induce the increases in cellularity or sizes of glomeruli in the kidney of B/Atg7^–/–^ mice. Glomerulonephritis activity score was 8.3 ± 2.8 versus 2.4 ± 1.8 for wild type and B/Atg7^–/–^ mice, respectively. These results suggest that autophagy deficiency in B cells suppress pristane-induced glomerulonephritis. We also examined lungs from mice treated with pristane for signs of lupus. Pulmonary hemorrhage has been shown in SLE patients and mice induced with pristane-induced lupus (69). We observed extensive perivascular and peribronchial infiltration in wild type but not autophagy-deficient mice at 6 months after pristane injection (**Figure 3B**). These results suggest that autophagy deficiency in B cells suppress pristane-induced autoimmune manifestations of lupus.

Autoreactive B cells can lead to over-production of antibodies and the deposition of antibody-immune complexes in the kidney, which can be the cause of glomerulonephritis. We therefore examined immune complexes in the kidneys of pristane-treated mice. As expected, we observed significant IgG deposition in the glomeruli of kidneys in wild type mice treated with pristane (**Figure 3C**). In contrast, IgG deposition was significantly reduced in B/Atg7^–/–^ mice (glomerulonephritis deposition score was 2.3 ± 0.5 versus 0.8 ± 0.6 for wild type and B/Atg7^–/–^ mice, respectively) (**Figure 3C**). Consistent with this finding, complement C3 staining were also dramatically reduced in B/Atg7^–/–^ mice when compared with wild type mice (glomerulonephritis deposition score was 2.2 ± 0.6 versus 0.7± 0.3) (**Figure 3D**), suggesting decreased complement activation in the absence of autophagy in B cells. These data support the conclusion that autophagy deficiency in B cells prevents autoimmune manifestations in the pristane-induced lupus model.

### Induction of Autoreactive Memory B Cells Depends on Autophagy

**Figure 4:**
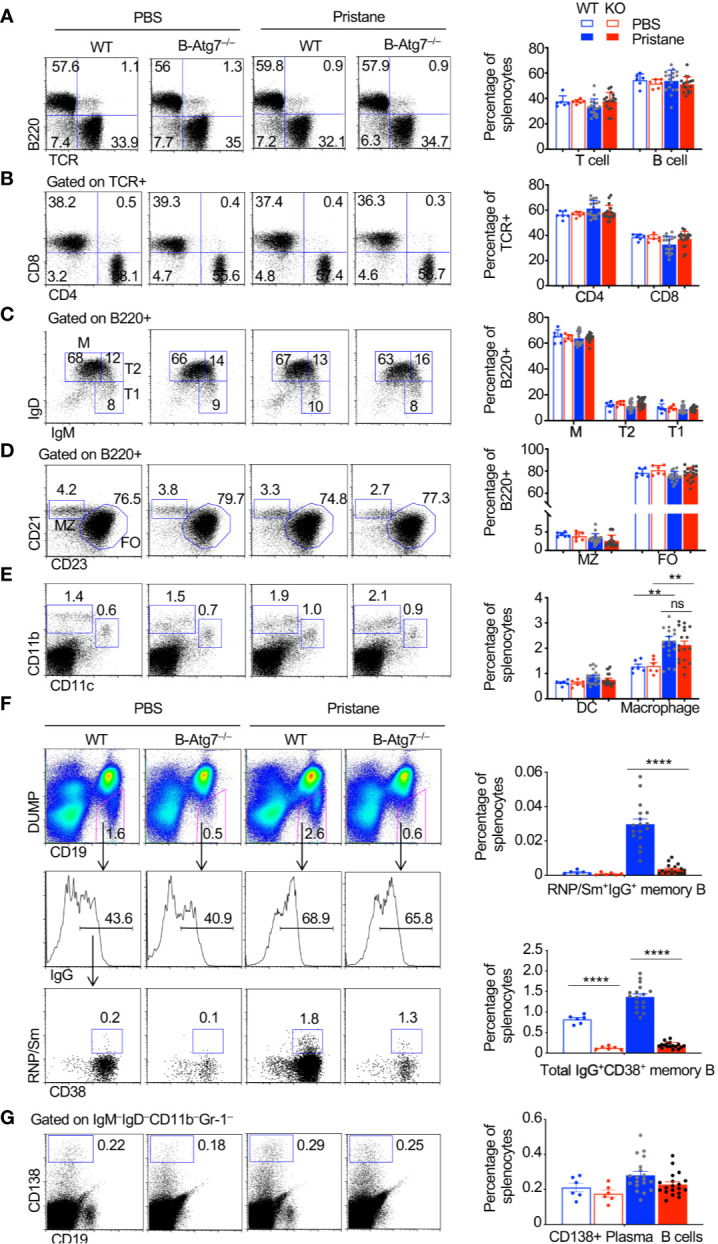
Accumulation of autoreactive memory B cells in WT but not B-Atg7^–/–^ mice injected with pristane. **(A–E)** Splenocytes from pristane or PBS injected WT or B/Atg7^–/–^ mice (6-month post injection) were stained with different fluorochrome-conjugated antibodies for T, B, dendritic cells (DC), and macrophages as indicated, followed by flow cytometry analysis. **(F)** Splenocytes were also stained with PE-conjugated antibodies to CD11b, Gr-1, IgM, IgD, and CD138; PE-Cy7-anti-CD19; Pacific Blue anti-CD38; APC-anti-IgG, and Alexa Fluor 488-conjugated RNP/Sm. DUMP^-^(IgM^-^IgD^-^CD11b^-^Gr-1^-^CD138^-^) CD19^+^ B cells were gated. Frequencies of RNP/Sm-specific memory B cells (DUMP^-^CD19^+^CD38^+^RNP/Sm^+^IgG^+^) and total IgG+ memory B cells (DUMP^-^CD19^+^CD38^+^IgG^+^) were analyzed by flow cytometry. **(G)** Splenocytes were stained with biotinylated antibodies to CD11b, Gr-1, IgM, IgD followed by Streptavidin PE-Cy7 and FITC-anti-CD19. IgM^-^IgD^-^CD11b^-^Gr-1^-^ cells were gated. CD138^+^CD19^lo/-^ plasma cells were then analyzed. A representative analysis of one mice/group was shown. Percentage of each cell population in the spleen was plotted. Data are presented as mean ± s.e.m. **P < 0.01, ****P < 0.0001, ns, not statistically significant, as determined by two-tailed Student’s *t*-test.

We next determined whether autophagy deficiency in B cells affects the composition and activation of different cell types in the immune system. We did not detect significant changes in the numbers and composition of T cells in B/Atg7^–/–^ mice compared to the wild type after the pristane treatment (**Figures 4A, B**). The number of B cells and the composition of mature B cells, as well as the transitional T1 and T2 cells, marginal zone (MZ) and follicular (FO) B cells (70) were also not significantly changed between wild type and B/Atg7^–/–^ mice at six months after pristane administration (**Figures 4A, C, D**). CD11c^+^CD11b^+^ dendritic cells and CD11c^–^CD11b^high^ macrophages, which can phagocytose pristane, were increased after pristane injection (**Figure 4E**). However, their percentages were comparable between wild type and B/Atg7^–/–^ mice (**Figure 4E**).

We next determined whether autoreactive memory B cells were present after treatment with pristane. Switched memory-like B cells (CD19^+^CD138^–^IgM^–^IgD^–^) were reported to be significantly increased in the pristane-treated BALB/c mice (71). However, whether autoantigen-specific memory B cells are expanded after pristane treatment have not been characterized. We also detected increased number of total IgG^+^ memory B cells (CD19^+^DUMP^–^CD38^+^IgG^+^) in wild type mouse after pristane treatment (**Figure 4F**). Those IgG^+^ memory B cells were significantly reduced in B/Atg7^–/–^ mice (**Figure 4F**). Moreover, we found that RNP/Sm-specific memory B cells (CD19^+^DUMP^–^CD38^+^ IgG^+^RNP/Sm^+^) in wild type mice could be detected by flow cytometry after injection with pristane (**Figure 4F**). These autoreactive B cells mainly display the DUMP^–^IgG^+^CD38^+^ memory but not the DUMP^–^IgG^+^CD38^–^GC B cell phenotypes (**Figure 4F**). Moreover, such autoreactive memory B cells were significantly reduced in B/Atg7^–/–^ mice (**Figure 4F**). These results suggest that autophagy is important for the development of both autoreactive memory B cells and nonautoreactive memory B cells. We also examined the IgM^–^IgD^–^CD19^lo^CD138^+^ plasma cells (72) in the spleens of pristane-treated mice. We observed a slight decrease in splenic plasma cells in pristane treated B-Atg7^–/–^ mice, but it did not reach statistical significance (**Figure 4G**). It is possible that a continuous replenishment of new short-lived plasma cells induced by pristane treatment compensates the loss of those cells in the absence of autophagy.

### Adoptive Transfer of Autoreactive Memory B Cells Restores Autoantibody Production in B/Atg7^–/–^ Mice

**Figure 5:**
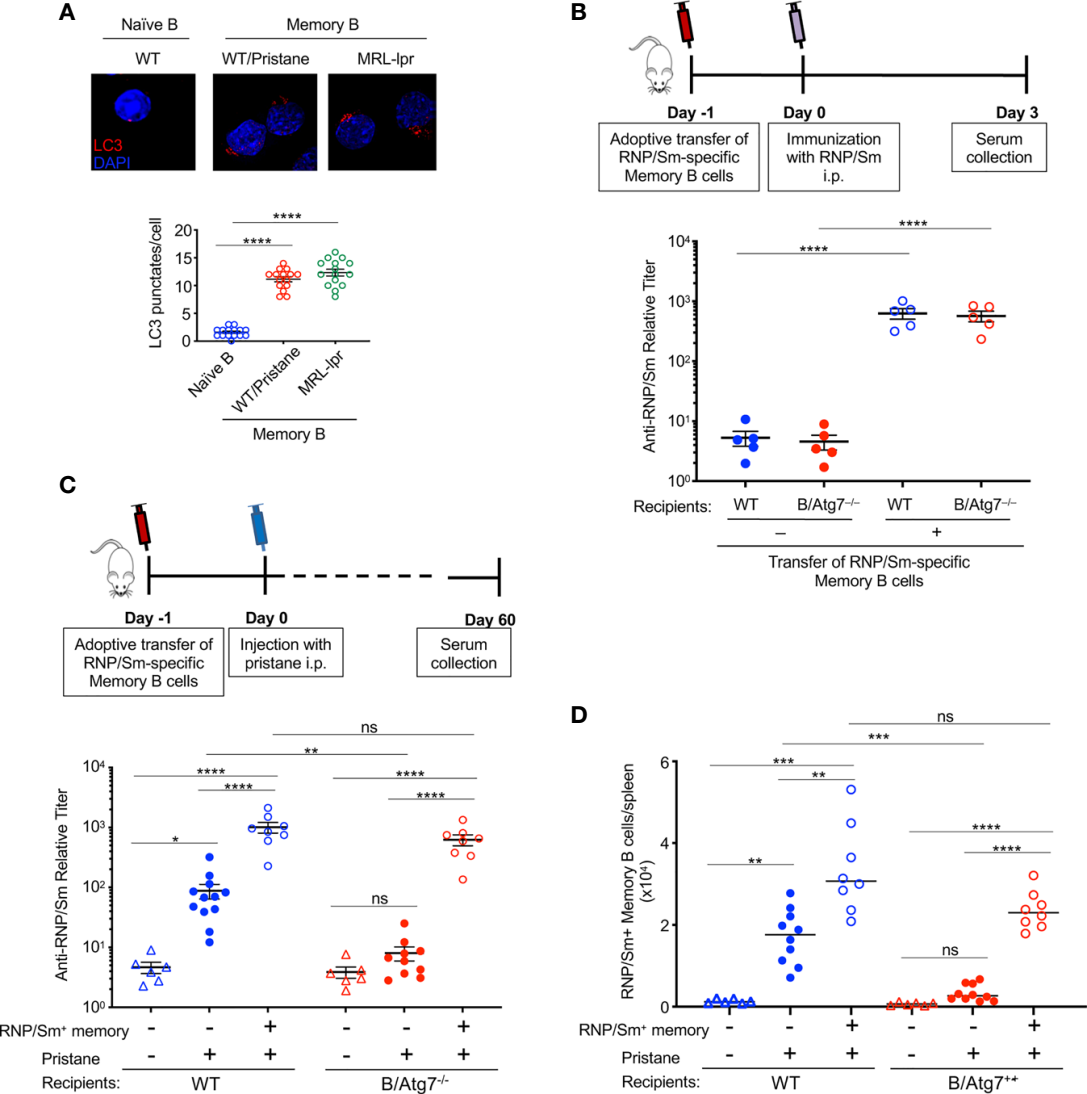
Transfer of autoreactive memory B cells rescues autoantibody production in B-Atg7^–/–^ mice. **(A)** RNP-specific memory B cells sorted from MRL-*lpr* mice or pristane-treated wild type mice, together with naïve B cells sorted from untreated wild type mice were used for immunocytochemistry staining of LC3. Data are representative of two independent experiments using cells from a pool of 3 mice each group for sorting. LC3 punctates per cell were quantitated (n=15). **(B)** RNP/Sm-specific memory B cells were sorted from pristane-treated wild type mice (5-6 mice post injection) and transferred (1x10^4^ cells/mice) retroorbitally into wild type and B-Atg7**^–/–^** mice. Mice were injected with RNP/Sm precipitated in Alum (20μg/mice, i.p.) a day later. Sera were collected 3 days after immunization and titers of RNP/Sm-specific antibodies were determined by ELISA. **(C, D)** RNP/Sm-specific memory B cells were sorted and transferred retroorbitally into WT and B-Atg7**^–/–^** mice (10^4^ cells/mice), followed by injection of pristane (0.5 ml, i.p.) one day later. Mice that did not receive memory cell transfer were included as controls. Sorted naïve B cells (2x10^5^/mice) were co-injected as filler cells to minimize cell loss during injection. Sera were collected 2 months later, and RNP/Sm specific antibodies were measured by ELISA **(C)**. DUMP^–^CD19^+^CD38^+^RNP/Sm^+^IgG^+^ memory B cells were also quantified **(D)**. Data are presented as mean ± s.e.m. (n=6-12). *P < 0.05, **P < 0.01, ***P < 0.001, ****P < 0.0001 (determined by two-tailed Student’s *t*-test). NS, not statistically significant.

We found that RNP^+^ memory B cells from pristane-treated wild type mice upregulated autophagy as shown by the increased LC3 punctate staining, to a similar extent as RNP^+^ memory B cells harvested from MRL-*lpr* lupus mice did (**Figure 5A**). To confirm the RNP^+^ memory B cells that we detected by flow cytometry (**Figure 4F**) could lead to autoantibody production, we sorted RNP/Sm-specific memory B cells from pristane-treated wild type mice and transferred them into wild type or B/Atg7^–/–^ recipient mice. We then challenged recipients of memory B cells and nonrecipient controls with RNP/Sm in Alum. Three days later, sera were collected to measure the production of RNP/Sm autoantibody. We detected production of RNP/Sm autoantibody in both wild type and B/Atg7^–/–^ recipient mice, suggesting CD19^+^DUMP^–^CD38^+^IgG^+^RNP/Sm^+^ autoreactive memory B cells are functional RNP/Sm-specific memory B cells and can be rapidly activated to develop into autoantibody-producing cells (**Figure 5B**).

Next, we asked whether RNP/Sm-specific memory B cells could restore pristane-induced autoantibody production in B/Atg7^–/–^ mice. RNP/Sm-specific memory B cells from pristane-treated wild type mice were sorted, and adoptively transferred into wild type or B/Atg7^–/–^ recipient mice in parallel experiments. After 24 h, both the recipient mice and control mice without receiving cell transfer were challenged with pristane. Atg7^–/–^ RNP/Sm-specific memory B cells from B/Atg7^–/–^ mice were not used for transfer because not sufficient number of cells could be obtained. Two months after pristane administration, autoantibodies against RNP/Sm could be detected in some wild type mice but not B/Atg7^–/–^ mice without receiving cell transfer (**Figure 5C**). However, adoptive transfer of wild type RNP/Sm-specific memory B cells resulted in significant production of RNP and Sm-specific antibodies in both wild type and B/Atg7^–/–^ recipient mice two months post pristane treatment (**Figure 5C**). Flow cytometry analysis of memory B cells indicate those transferred RNP/Sm-specific memory B cells were expanded in the spleens of B/Atg7^–/–^ recipient mice compared with non-recipient controls (**Figure 5D**). These results suggest that autoreactive memory B cells can restore autoantibody production in B/Atg7^–/–^ mice. These results suggest that autophagy plays an important role in the protection of autoreactive memory B cells to maintain autoantibody production in lupus.

## Discussion

Significant expansion of memory B cells has been reported to be present in humans and mice with lupus. The contribution of the expanded memory B cell population to lupus pathogenesis is unclear. Here we show that autoantigen-specific memory B cells can be detected in *lpr* mice, and in mice with pristane-induced lupus. Moreover, these autoreactive memory B cells increased autoantibody production after adoptive transfer. Interestingly, deficiency of autophagy led to the loss of autoreactive memory B cells and attenuated glomerulonephritis and pulmonary inflammation in a pristane-induced lupus model. These results suggest that autoreactive memory B cells are important for the maintenance of autoantibody production and autoimmune manifestations in lupus. Autophagy plays an important role in promoting autoantibody production by protecting these autoreactive memory B cells.

Previous studies have observed that switched memory B cells were increased in MRL-lpr lupus mice, however, the identity of those memory B cells has not been well characterized. Using fluorochrome-conjugated autoantigens, we detected IgG^+^CD38^+^ memory B cells with specificity for different autoantigens in mice with lupus by flow cytometry. We have demonstrated that autoantigen-specific memory B cells such as Sm/RNP or dsDNA specific autoreactive memory B cells are increased in MRL-lpr lupus mice. We have also found that Sm/RNP specific autoreactive memory B cells are increased in mice with pristane-induced lupus. Like memory B cells specific for foreign antigens that depend on autophagy for survival (30), these autoreactive memory B cells also upregulated expression of autophagy-related genes and display high levels of autophagy. Deletion of autophagy leads to the loss of autoreactive memory B cells and total IgG^+^ memory B cells in pristane-treated mice to a similar extent, suggesting an equally critical role for autophagy in the protection of autoreactive memory B cells and normal memory B cell compartment. We have previously found that autophagy is essential for the long-term survival of memory B cells but not the generation of memory B cells after their initial formation (30, 31). We and others have also shown that autophagy is not required for GC formation and B cell activation or proliferation (30, 31, 38). It is thus possible that autoreactive memory B cells are generated in normal numbers initially but cannot persist in autophagy-deficient mice.

It has been shown that knockout of Atg5 in B cells attenuates the development of autoantibody production, lymphocyte infiltration and mortality in toll-like receptor 7-transgenic mice (40), supporting an important role for B cell autophagy in the development of lupus-like autoimmunity. In addition, autophagy is important for the survival of long-lived plasma cells and autoantibody production during autoimmune responses in *lpr* mice (38). In the current study, we show that autoreactive memory B cells specific for self-antigens can be induced in a pristane-induced mouse lupus model, but these cells are significantly reduced in autophagy-deficient mice. Adoptive transfer of autoreactive memory B cells led to autoantibody production in recipient mice. Moreover, transfer of wild type autoreactive memory B cells restored autoantibody production in autophagy-deficient mice upon pristane injection. Our data thus support an important role for autoreactive memory B cells in the development of lupus by promoting persistent autoantibody production. Although our study has demonstrated the important contribution of memory B cells to autoantibody production, we are not excluding the role of autophagy in other B cell types, particularly long-lived plasma cells in autoantibody production and immune complex deposition. Taking these data together, autophagy is likely required for the survival of both autoreactive memory B cells and autoreactive long-lived plasma cells to promote autoantibody production.

Treatment with pristane can induce glomerulonephritis with enlargements of kidney glomeruli, the accumulation of intraglomerular mesangial cells and deposits of immune complexes in the kidney. We observed that autophagy deficiency in B cells resulted in the loss of autoreactive memory B cells, reduced immune complex and complement deposition and ameliorated the development of autoimmune symptoms induced by pristane, including the glomerulonephritis and pulmonary capillaritis. These results suggest that accumulation of autoreactive memory B cells, which could lead to overproduction of autoantibodies, is potentially the main causes of kidney and lung lesions in the pristane-induced lupus model. Significant expansion of memory B cells in systemic autoimmune diseases also supports the important roles for autoreactive memory B cells in disease development.

Pristane-induced lupus can cause pulmonary hemorrhage resembling that in SLE, and cause mortality in mice on C57BL/6 background (52, 53). Our results demonstrate that loss of ATG7 in B cells reduced the susceptibility of mice to pristane-induced pulmonary hemorrhage. While treatment with pristane caused 18% death in wild type mice, B/Atg7^–/–^ mice were resistant. Moreover, six months after pristane injection, B/Atg7^–/–^ mice did not show significant pulmonary capillaritis or leukocyte infiltration compared to wild type controls. B cells have been reported to be required for pristane-induced pulmonary hemorrhage (52, 53, 67). Since memory B cells are potent antigen presenting cells and autophagy could regulate antigen presentation (16, 17, 73), it is possible that defective antigen presentation by autophagy-deficient memory B cells resulted in defective amplification of autoimmune responses, thus leading to the reduced susceptibility of B/Atg7^–/–^ mice to pristane-induced pulmonary hemorrhage. Alternatively, reduced autoantibody production and immune complex deposits in B/Atg7^–/–^ mice could significantly attenuate pristane-induced pulmonary vasculitis. These possibilities are not mutually exclusive and remain to be investigated.

Although adoptive transfer of wild type Sm/RNP specific autoreactive memory B cells restored autoantibody production in B-Atg7^–/–^ mice, we did not observe significantly increased lung and kidney pathology in pristane-treated B-Atg7^–/–^ mice two months after adoptive transfer (data not shown). Several mechanisms may account for this observation. First, it is possible that multiple different types of autoreactive memory B cells are needed for producing lupus pathology in kidney and lung in pristane-treated B-Atg7^–/–^ mice, while transfer of Sm/RNP specific memory B cells alone is not sufficient. Second, a larger number of Sm-specific memory B cells may be required for transfer into B-Atg7^–/–^ mice to produce pristane-induced lupus pathology, and it could take longer than 2 months for recipients to develop lupus pathology in kidney and lung. Third, besides protecting memory B and plasma cell survival, autophagy may have additional roles in B cells which is also required for producing lupus pathology in pristane-treated mice. The exact mechanism leading to this phenomenon remains to be investigated.

Multiple different studies have confirmed an essential role for autophagy in the maintenance of long-lived plasma cells (35, 36, 38–40). The effect of autophagy on short-lived plasma cells has been less clear. While autophagy has been shown to be required for B cell differentiation into ASCs *in vitro*, several studies have shown that plasma cell number in the spleen is not affected by the deletion of Atg5 in B cells (36, 38). We have observed a slight decrease in the splenic CD19^lo^IgM^–^IgD^–^CD138^+^ plasma cells in pristane treated B-Atg7^–/–^ mice, but it did not reach statistical significance (**Figure 4G**). Interestingly, it has also been shown that autophagy deficiency does not affect short-lived plasma cells in the spleen of autoimmune CD21creAtg5^f/f^ lpr/lpr mice (38). A possible explanation is that a continuous replenishment of new short-lived plasma cells induced by pristane treatment in our model compensates the loss of those cells in the absence of autophagy. Another explanation is that autophagy might be differentially required for the long-lived and short-lived plasma cell survival. Long-lived, slowly dividing plasma cells in the bone marrow might rely more on autophagy for their survival, while short-lived plasma cells rely less on autophagy for their survival.

**Figure 6:**
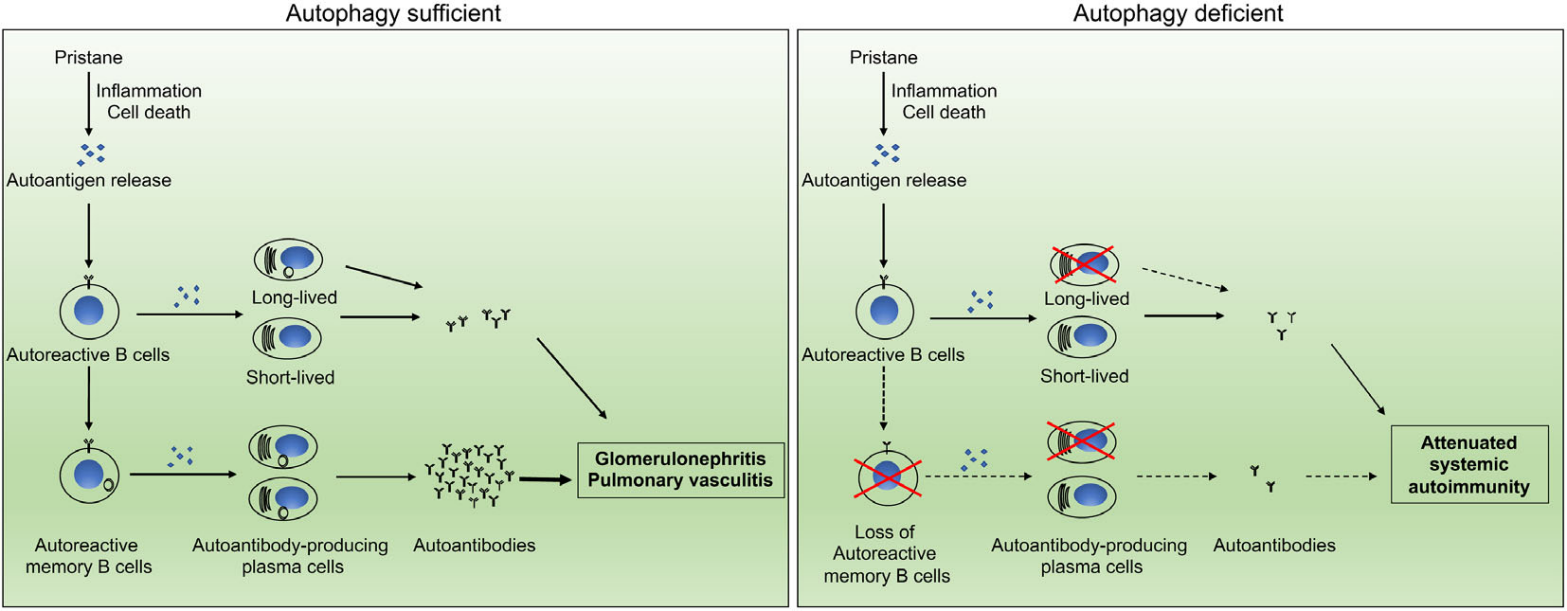
Schematic representation of the impact of autophagy on autoantibody production. Autophagy regulates the development of systemic autoimmunity through regulation of autoreactive memory B cells and long-lived plasma cells in pristane-induced lupus model.

Overall, humoral immune memory is mediated by long-lived plasma cells, as well as memory B cells which upon antigenic challenge can develop into plasma cells. We propose a model for autophagy in B cells in the regulation of pristane-induced lupus (**Figure 6**). Pristane treatment induces profound inflammation and apoptotic cell death (52, 71, 74), which led to breakdown of tolerance and activation of autoreactive B cells *via* T cell dependent or independent mechanisms. Some of the activated autoreactive B cells develop into short-lived or long-lived plasma cells which produce autoantibodies against RNP, Sm and other nuclear autoantigens, whereas some of the activated autoreactive B cells will give rise to autoreactive memory B cells. The persistent presence of autoantigens in pristane-treated mice results in continual stimulation of autoreactive memory B cells which can rapidly differentiate into long lived autoantibody-producing plasma cells. The rapid development of memory B cells into antibody-producing cells accelerates autoantibody production and sustains systemic autoimmunity in pristane-treated wild type mice. The loss of autophagy in B cells abolishes the survival of memory B cells and long-lived plasma cells, resulting in significant reduction in autoantibody production and attenuated glomerulonephritis and pulmonary inflammation in the pristane-treated B/Atg7^–/–^ mice (**Figure 6**).

Memory B cells specific for autoantigens has not been demonstrated in previous studies. Whether an essential role for autophagy in the persistent of these autoantigen-specific memory B cells has not been established. Our current study shows the existence of memory B cells specific for Sm/RNP and dsDNA in *lpr* mice by flow cytometry. Moreover, Sm/RNP-specific memory B cells were reduced in the pristane-induced autoimmune lupus model. Therefore, our study demonstrates the role for autophagy in the maintenance of autoimmune memory B cells specific for defined autoantigens. In vitro differentiation and adoptive transfer experiments suggest that these memory B cells are functional autoreactive memory B cells and can develop into autoantibody-producing cells. Deficiency in autophagy reduced autoreactive memory B cells and attenuated the autoimmune manifestations in pristane-treated mice. However, transfer of wild type autoreactive memory B cells could restore autoantibody production in autophagy-deficient mice. These data suggest that autoreactive memory B cells plays an important role in the development of humoral autoimmunity. Our study, together with the previous report that autophagy is required for long-lived plasma cells in *lpr* mice (38), suggests that targeting autophagy may be effective for eliminating autoreactive memory B cells and long-lived plasma cells to control autoimmunity in lupus.

## Data Availability Statement

The raw data supporting the conclusions of this article will be made available by the authors, without undue reservation.

## Ethics Statement

The animal study was reviewed and approved by Institutional Animal Care and Use Committees of Baylor College of Medicine and the Houston Methodist Research Institute.

## Author Contributions 

AJ and MC designed and performed experiments and analyzed data. RS, YF, and JMW performed experiments. JW designed experiments and wrote the manuscript. MC supervised the study and wrote the manuscript. All authors contributed to the article and approved the submitted version.

## Funding

This study was supported by funding from American Heart Association (15GRNT25700357 to MC), Lupus Research Institute (MC and JW), Department of Defense (PR140593 to MC), and the NIH (R01AI116644 and R01AI123221 to JW). Flow cytometry and cell sorting for this project was supported by the Cytometry and Cell Sorting Core at Baylor College of Medicine with funding from the NIH (P30 AI036211, P30 CA125123, and S10 RR024574).

## Conflict of Interest

The authors declare that the research was conducted in the absence of any commercial or financial relationships that could be construed as a potential conflict of interest.
